# Tunning of Templated CuWO_4_ Nanorods Arrays Thickness to Improve Photoanode Water Splitting

**DOI:** 10.3390/molecules26102900

**Published:** 2021-05-13

**Authors:** Nasori Nasori, Dawei Cao, Zhijie Wang, Ulya Farahdina, Agus Rubiyanto, Yong Lei

**Affiliations:** 1 Group of Applied Nanophysics (Fachgebiet Angewandte Nanophysik), Institute of Physics & IMN MacroNano®, ZIK Technical University of Ilmenau, 98693 Ilmenau, Germany; 2 Department of Physics, Faculty of Sciences and Data Analytic, Sepuluh Nopember Technology Institute, Surabaya 60111, Indonesia; ulyafarahdina06@gmail.com (U.F.); agus.rubi65@gmail.com (A.R.); 3 Department of Physics, Faculty of Sciences, University of Jiangsu, Zhenjiang 212013, China; dwcao@ujs.edu.cn; 4 Semiconductor Materials Science Key Laboratory, Semiconductors Institute, Chinese Sciences Academy, Beijing 100083, China; wangzj@semi.ac.cn

**Keywords:** n-type CuWO_4_, nanorod, photoelectrochemical, sustainable energy, water splitting

## Abstract

The fabrication of the photoanode of the n-type CuWO_4_ nanorod arrays was successfully carried out through electrochemical deposition using anodic aluminum oxide (AAO) control templates and for the first time produced distinct gaps between the nanorod arrays. The effectiveness and efficiency of the resulting deposition was shown by the performance of the photoelectrochemical (PEC) procedure with a current density of 1.02 mA cm^−2^ with irradiation using standard AM 1.5G solar simulator and electron changed radiation of 0.72% with a bias potential of 0.71 V (vs. Ag/AgCl). The gap between each nanorod indicated an optimization of the electrolyte penetration on the interface, which resulted in the expansion of the current density as much as 0.5 × 10^24^ cm^−3^ with a flat band potential of 0.14 V vs. Ag/AgCl and also a peak quantum efficiency of wavelength 410 nm. Thus, also indicating the gaps between the nanorod arrays is a promising structure to optimize the performance of the PEC water splitting procedure as a sustainable energy source.

## 1. Introduction

The world is always in need of energy; however, important nonrenewable energy sources have decreased in recent decades. As a result, researchers have been urged to grow new, sustainable, and environmentally friendly energy sources. In 1972, Fujisima and Honda initiated significant progress on inexpensive and renewable energy technology based on the ecofriendly TiO_2_ that successfully used solar power [1]. In addition, several exploitations and explorations of many other materials [2], structural repair [3], as well as fabrication techniques have been carried out to increase the performance and quality of a wide range of solar power transformation conversion tools [4,5]. One that is worth examining is the creation of hydrogen fuel using the PEC water splitting procedure that has been proven to be affordable and eco-friendly. Gratzel et al. [6] along with other researchers [7,8] have realized effective materials such as BiFe_2_O_3_, CuBiO_4_, CuWO_4_, as well as others in the form of multilayer thin films for the application of PEC. However, the numerous layers in these films impede the viability of the application of PEC due to the fact that it must fit the bandgap (in the effective range between 1.6 eV up to 3.2 eV) and also the fabrication and deposition of the materials within a film significantly determines the quality and outcome of mobility, electron majority (type n), hole majority (type p), optical absorption, and adequate resistance.

Tungstate oxide (WO_3_) is a fascinating substance to investigate since it has high potential in the fields of optical, optoelectronic, diamagnetic, photocatalyst, and sensor [9,10,11]. One particular model for tungstate is copper tungstate oxide (CuWO_4_). To date, most of the publications on CuWO_4_ thin film using a bandgap in the ranges of 1.8 eV up to 2.4 eV are feasible to be used as the main material for the application of PEC by using various techniques of deposition, as shown by Martin et al. [12], that reported the deposition of WO_4_ on Cu. Despite the many publications on CuWO_4_ thin films, there has been no publication that has reported on obtaining the theoretical CuWO_4_ efficiency value of 13%.

The creation of electrodes is thicker through the process of sol–gel or electrodeposition and causes a low external quantum efficiency [13]. Moreover, balanced development in CuWO_4_ nanostructure is assumed to increase the efficiency of light immersion and expand the contact surface zone, thus improving the performance of PEC. Despite the capacity of CuWO_4_ in efficiently transferring holes on the interface of the electrolyte–material by diffusing in an axial direction, the small electron mobility of CuWO_4_ impedes the viability of transferring electrons in a radial direction to the current collector [14]. Nanoarrays are an alternative to overcome this problem because the core materials that form these nanoarrays can effectively function as electron separators and transport the current in the axial direction [13]. This novel approach of fabricating CuWO_4_ in nanostructure form aims to increase the efficiency of visible light absorption (UV–Vis) and the stability of the material. On the other hand, the fabrication of this material has a sequential and complex multistep process and requires a qualitative optimization approach toward the current of the charge carrier in forming the nanostructure [15].

This research has successfully fabricated CuWO_4_ nanorod arrays through electrodeposition of CuWO_4_ using nanoimprinted AAO patterns with a constant current of 0.4 mA cm^−2^. The imprinted AAO template is a simple technology that involves an electrochemical process in the formation process; however, it is relatively cheaper by the anodizing process so that the width and depth of the imprinted template AAO are easy to control, which makes this technology able to produce various nanostructures [16]. By utilizing the control of imprinted AAO templates, distinct gaps between the CuWO_4_ nanorod arrays are obtained and used as a photoanode for the PEC water splitting procedure. The simulation of electric field distribution on CuWO_4_ has been carried out in previous research [17]. The resulting CuWO_4_ nanorod arrays were then tested using a solar simulator (100 mW cm^−2^) to measure and compare the electrode performance and the theoretical efficiency between CuWO_4_ nanorod arrays that possess distinct gaps between the arrays and CuWO_4_ thin films.

## 2. Materials and Methods

### 2.1. Materials and Chemicals

Copper(II) nitrate trihydrate (Cu(NO_3_)_2_.nH_2_O) (98%), peroxotungstic acid (H_2_W_2_O_11_) (95%), nickel(II) sulfate (NiSO_4_) (98%), nickel(II) chloride (NiCl_2_) (98%), boric acid (H_3_BO_3_) (97%), sodium hydroxide (NaOH) (99%), titanium tetrachloride (TiCl_4_) (99%), phosphoric acid (H_3_PO_4_) (70%), sodium sulfate (Na_2_SO_4_) (≥99%), copper(II) chloride (CuCl_2_) (98%), and acetone (C_3_H_6_O) (99.8%) were utilized as obtained from Sigma Aldrich. The Loctite 3430 insulating epoxy was used. Thick Al foil (99.99%) and Copper Cu tape (AT528) was acquired from Advance Tapes., while the TEC-15 fluorine-doped tin oxide (FTO) glass (density: 150 nm/1.1 mm; resistivity of sheet: ≤20 Ω/cm^2^, transmissivity: 88.9% wavelength 400 nm) was bought from NGS Glass.

### 2.2. Manufacture of Ni Nanopore Arrays

The procedure of creating the pores from the Al nickel (Ni) pore arrays sheet is pressed in order to obtain a round shape with a diameter of 3 cm, then washed cleaned with acetone (99%) in ultrasonic and DI water for 30 min. Furthermore, form Al polishing with the electrochemical method (with applied voltage 30 V) by using a solution of HClO_4_ and ethanol with a solution ratio of 1:7 accordingly. Then, Once the Ni sheet stem is placed on the Al, which, after polishing using a pressure of 3 kPa for 3 min, will produce a regular pattern on the Al surface. The anodization procedure was carried out at a temperature of 4 °C for 1 h with a voltage of 160 V. This is known as the alumina anodization process of oxide or AAO template forming. Then, to expand the AAO pores following the anodization procedure, the AAO template was submerged in a solution of 5 wt% H_3_PO_4_. This submerging process was carried out to control the width of the gap of the AAO template by varying the length of time it was submerged in the solution, which, in turn, produces different width of the gap. The next step was to clean and dry the imprinted AAO template before Au deposition in the range of 20 nm up to 25 nm using an electron beam on the Au at a minimal vacuum condition of 10^−3^ Torr.

### 2.3. CuWO_4_ Synthetics

All reactors were purchased and used without further purification. The materials used were the materials used by Gaillard et al. [18], which consisted of a composition of 25 mM Cu(NO_3_)_2_.nH_2_O and 25 mM H_2_W_2_O_11_. Peroxytungstate solution was obtained by dissolving tungsten metal powder into 10 mL dionixide (DI) water, which consisted of 30% part hydrogen peroxide. Then, as much as 25 mM of these two base solutions were blended in 70:30 DI water: 2-propanol proportion and finished by utilizing 10% nitric acid solution to control the pH of the solution in its final state.

### 2.4. CuWO_4_ Nanorod Arrays, and Thin Films Deposition

CuWO_4_ nanorod arrays was deposited over the AAO template preparation, which were covered by TiO_2_/Au and CuWO_4_ thin films on FTO glass by utilizing constant current applied electrodeposition ‒1 mA versus Ag/AgCl for an hour at 30 °C (situ controller) by utilizing Biologic channel. This preparation was then heated at 550 °C for 2 h with an MTI furnace box and was left until arriving at room temperature. The subsequent stage was Ni deposition (0.38 M NiSO_4_, 0.12 M NiCl_2_, and 0.3 M H_3_BO_3_) by electrodeposition with constant applied potential −10 V for 3 h, accompanied by discarding the posterior layer with CuCl_2_ solution (26.21 g Cu_2_SO_4_, 36 mL HCl, and 700 mL DI water). To exclude the AAO template, 10 wt% H_3_PO_4_ was used at 30 °C for 2 h. To secure CuWO_4_ nanocore arrays, TiO_2_ was deposited with atomic layer deposition (a Picosun Sunale R150 ALD reactor) on 5 nm density at 200 °C. The titanium dioxide had been deposited by utilizing titanium (IV) chloride (TiCl_4_) and distilled water (H_2_O) as Ti and O forerunners, respectively. Then, TiO_2_ deposition was completed at 200 °C as well as a common cycle comprised TiCl_4_-N_2_ purge-H_2_O-N_2_ purge (1 cycle). The development pace of TiO_2_ was around 0.6 nm per cycle.

### 2.5. CuWO_4_ Characterizations and PEC Measurement

The energy-dispersive X-ray (EDX) mapping was observed on an S4800 HITACHI (Japan) field emission scanning electron microscope. The morphologies were obtained with a JEM-2100F transmission electron microscope (TEM) operated at an acceleration voltage of 100 kV. High-resolution transmission electron microscopy (HRTEM) images and the corresponding selected area electron diffraction (SAED) analysis were attained at an acceleration voltage of 200 kV. The X-ray diffraction (XRD) patterns were recorded on a Bruker D8 Advance diffractometer equipped with a graphite monochromatized high-intensity Cu Ka radiation (1.54178 Å). The UV–Vis absorption spectra were obtained using a Varian Cary 5000 UV–Vis–NIR spectrophotometer. The EQY was carried out under 150 W from a xenon lamp (controlled by TRACQ BASIC software and connected via Merlin radiometry digital lock-in system) inside a monochromator grating. Current–potential plots and impedance characterization were carried out using the digital BioLogic potentiostat (SP-200) and sodium sulfate with a concentration of 0.1 M as the solution. A Pt counter electrode and an Ag/AgCl reference electrode were used during the measurements.

## 3. Results and Discussion

**Figure 1:**
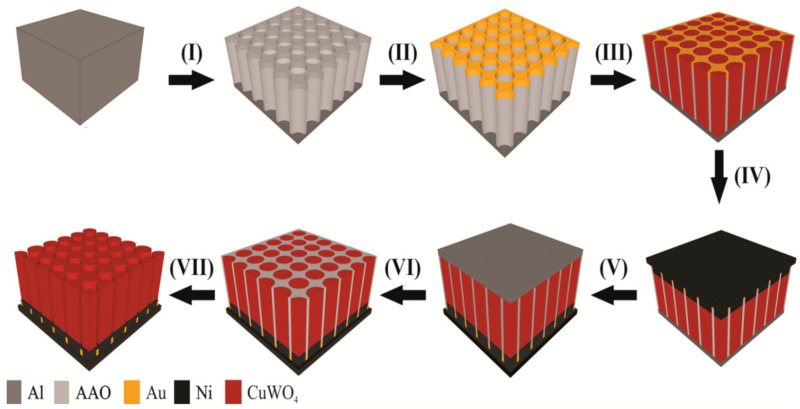
The sequential phases of the fabrication of the CuWO_4_ nanocore arrays using an imprinted AAO template with Ni sheet as a substrate to support the photoanode.

**Figure 2:**
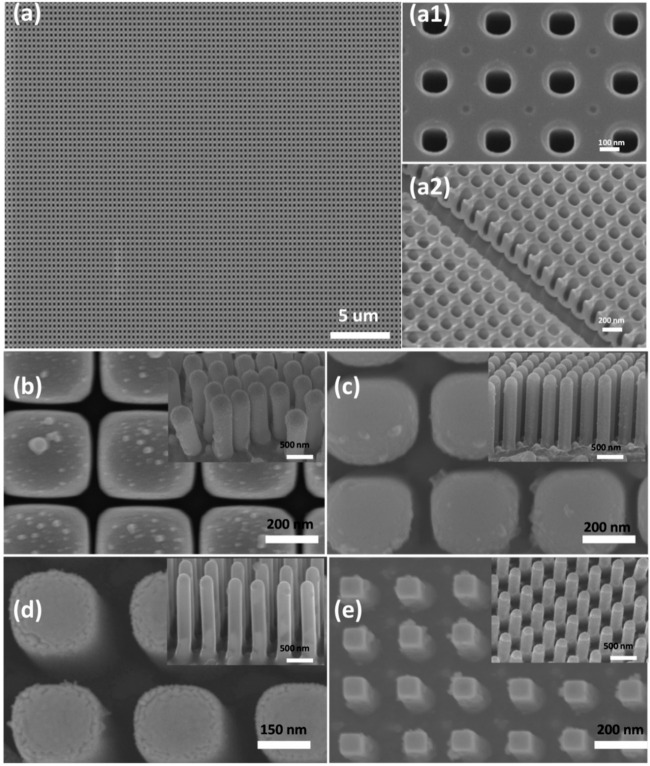
(**a**) SEM images of the top view of the imprinted AAO template with a highly uniform order; (**a1**) SEM images of the top view of the imprinted AAO template with a scale of 100 nm; (**a2**) a side view of the imprinted AAO template; and (**b**–**e**) SEM images of the photoanode of the CuWO_4_ nanorod arrays with an estimated gap between the CuWO_4_ nanorod arrays of 50 nm, 100 nm, 150 nm, and 200 nm, respectively.

To date, the nanoimprinted AAO template method transformed into one of the best advancements that have been demonstrated to significantly contribute to a decent fabrication of nanostructure arrays [19,20] and is also relatively low cost [21] and simple to construct [22]. Figure 1 shows a detailed schematic step-by-step fabrication of the CuWO_4_ nanorod arrays. The first step of this fabrication is using Ni sheet as the self-support on aluminum. The second step is anodization for as long as 30 min, submerged in an H_3_PO_4_ (5 wt%) solution with control pores between 100 nm and 200 nm. Figure 2a–c shows the SEM images of the AAO template and is an overview of the gap between two pores that is dependent on the length of time it was submerged in the H_3_PO_4_ (5 wt%) solution. The third step is the deposition of TiO_2_ and Au. The fourth step is electrodeposition using three electrodes with a potential bias of −0.4 V versus Ag/AgCl and annealing at a temperature of 500 °C for 2 h. The fifth step is nickel plating and the sixth step is the removal of the back side of the AAO template. The last step is the removal of the template by submerging in an H_3_PO_4_ (5 wt%) solution for 3 h and at a temperature of 30 °C. A TiO_2_ 5 nm coating layer is added to all the samples using atomic layer deposition (ALD).

The CuWO_4_ nanorod arrays obtained from AAO are shown in Figure 2d,e. It can be seen that the morphology of the CuWO_4_ nanorod arrays is well organized due to the high structure control of AAO. The length of the nanorod arrays that are relatively equivalent, in the range between 1 µm and 1.2 µm, and the relatively wide gap between two CuWO_4_ nanorod arrays will significantly affect the performance of PEC. Electrolyte penetration can easily work on CuWO_4_ nanorod arrays due to the absence of obstacles, resulting in a high hole and electron mobility. In addition, another advantage of AAO is the low contamination effect even when heated with CuWO_4_ at temperatures exceeding 550 °C. SEM images shown in Figure 2b,c clearly show no trace of AAO, which is proof that this way is very efficacious for the fabrication of this material and producing gaps between the CuWO_4_ nanorod arrays of 50 nm, 100 nm, 150 nm, and 200 nm, respectively.

**Figure 3:**
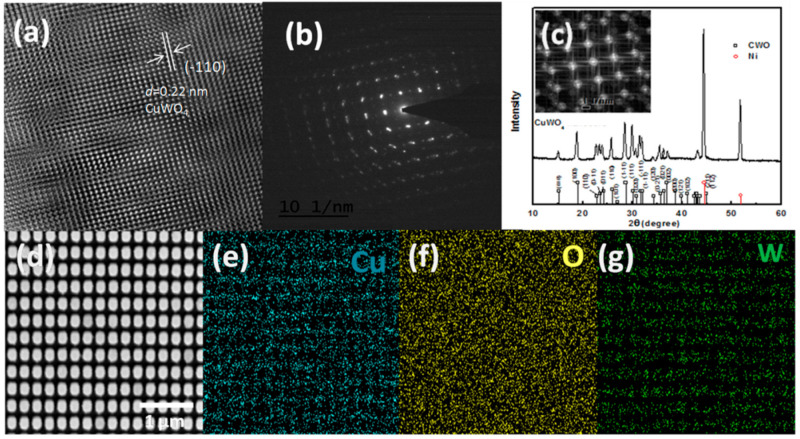
(**a**) High-resolution transmission electron microscopy (HRTEM) image of a sample of the photoanode of the CuWO_4_ nanorod arrays after deposition; (**b**) a selected area from the electron diffraction pattern of a sample of the photoanode of the CuWO_4_ nanorod arrays; (**c**) XRD pattern of a sample of the photoanode of the CuWO_4_ nanorod arrays (insert: SAED pattern of a sample of the photoanode of the CuWO_4_ nanorod arrays, the smaller image shows the inverse FFT cell); and (**d**) SEM images of the EDX mapping that represent (**e**) the Cu element, (**f**) the O element, and (**g**) the W element, respectively.

Figure 3a shows us the HRTEM images and SAED patterns of the CuWO_4_ nanorod arrays with a nanorod array structure diameter of 200 nm, similar to that of previously reported CuWO_4_ thin films. This is an important basis to propose a concept that irradiation can simply penetrate the surface and the gaps between the CuWO_4_ nanorod arrays, thus causing a hydrogen evolution reaction in the counter electrode, and at the same time, an oxygen evolution reaction occurs in the CuWO_4_ nanorod arrays. This is different from CuWO_4_ thin films in which irradiation only occurs on the surface. To make the results of this research more credible, in Figure 3b, a detailed electron diffraction tomography of the CuWO_4_ nanorod arrays is shown to obtain a standard structure with a *cmcm* space group and unit cell parameter of *a* = 3.72 Å, *b* = 5.51 Å, and *c* = 3.98 Å. A high-quality single-crystal appears in this image, as was also visible in the structure of previous research. Furthermore, also shown in Figure 3b, HRTEM using an aberration corrector on the projected crystal quality samples shows that the average gap that is formed between adjacent lattices is 0.22 nm, which in accordance with the area of the material (−110). This also gives information about the high crystallinity consistency, structure, and distribution of Cu and W within the CuWO_4_ nanorod arrays. Subsequently, complimentary information about the elements from the fast Fourier transform (FFT) simulation is clearly shown in Figure 3c, in which the atom O is randomly distributed in the structure that was formed. The XRD pattern of the electrode is shown in Figure 3c, along with the Miller index of the single-crystal structure. For comparison, measurements were performed to both CuWO_4_ thin films and CuWO_4_ nanorod arrays where both samples were heated in air at a temperature of 550 °C for 2 h, and as a result of this fabrication method, XRD patterns are formed on the material [11,19], where the area of the formed CuWO_4_ nanorod arrays material (001) is at a 24.21° angle and the area of the material (002) reflects an angle of 33.27°. To confirm the chemical composition of the CuWO_4_ nanorod arrays further, an energy-dispersive X-ray (EDX) mapping is carried out on the constituent particles and is shown in Figure 3e–g, based on the SEM image shown in Figure 3d, and by giving the sequential signal information of Cu, W, and O. The increased crystal quality may be due to the thin coating layer of TiO_2_ on the imprinted AAO templates that can supply an evenly distributed electric field and activation energy for electrochemical deposition to form the CuWO_4_ nanorod arrays. Additionally, the thin coating layer of TiO_2_ can be useful to reduce resistance. The composition from one of the CuWO_4_ nanorod array samples was identified using X-ray photoelectron spectroscopy (XPS) likewise to that depicted in standard SI W^6+^ [10]. The Cu (2p) spectrum region is shown by a Cu (2p _3/2_) peak and a Cu (2p _1/2_) at 933.8 eV and 954.9 eV, respectively, which is also known as the Cu^2+^ energy peak [12]. Lastly, the O area (1s) that is shown with one peak (1s) is the oxide lattice domain and is generally used to depict the surface of hydroxide on the surface of metal oxide.

**Figure 4:**
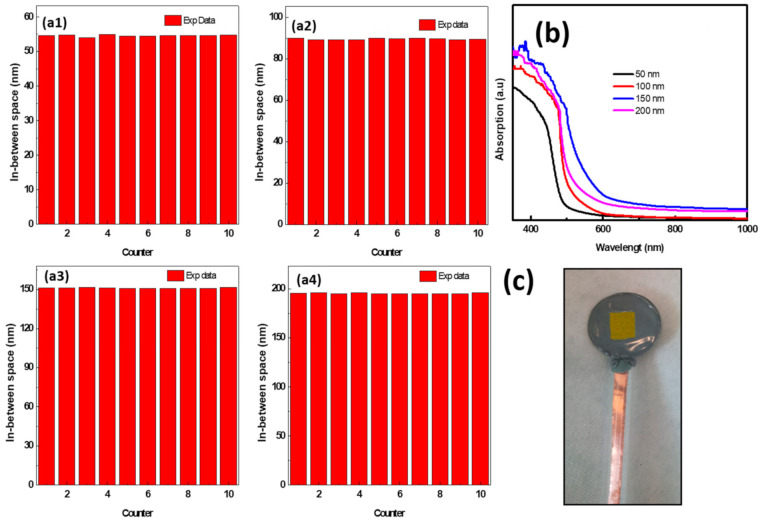
The average measurement of the samples of the photoanode of the CuWO_4_ nanorod arrays with distinct gaps between the nanorod arrays of (**a1**) 50 nm, (**a2**) 100 nm, (**a3**) 150 nm, and (**a4**) 200 nm; (**b**) the absorption measurement within the UV–Vis light range of each photoanode samples; and (**c**) image of the photoanode of the CuWO_4_ nanorod arrays covered with epoxy used for the electrical properties testing.

The distribution of the gaps between the CuWO_4_ nanorod arrays for 50 nm, 100 nm, 150 nm, and 200 nm is, respectively, shown in Figure 4a1–a4. It can be seen that for 50 nm the gaps between the CuWO_4_ nanorod arrays are in the range of 48 nm up to 54 nm (Figure 4a1), for 100 nm, the gaps are in the range of 94 nm up to 102 nm (Figure 4a2), for 150 nm, the gaps are in the range of 148 nm up to 152 nm (Figure 4a3), and for 200 nm, the gaps are in the range of 198 nm up to 202 nm (Figure 4a4). This shows that the nanoimprinted AAO template is an effective technology in controlling CuWO_4_ to become a nanostructure, which has never been performed previously. The result of the light absorber measurement using UV–Vis spectrophotometry on each distinct gap between the CuWO_4_ nanorod arrays is shown in Figure 4b. The results present an optimal condition that is obtained by each compartment, thus becoming the basis to claim an increased performance of the PEC water splitting procedure on these CuWO_4_ nanorod arrays. The light absorber measurement carried out on the distinct gaps between the CuWO_4_ nanorod arrays, in theory, provides a good chance to optimize the incoming light absorption ability of the photoanode. The absorption spectrum of the samples shown in Figure 4b, shows an increase in incoming light absorption on wavelengths in the range of 300 nm up to 620 nm, which is related to the antireflection characteristic of the CuWO_4_ nanorod array structure. Furthermore, the absorption spectrum of the CuWO_4_ nanorod arrays shown in Figure 4b presents absorption in the visible light range. Absorption tends to happen at around 500 nm for gaps between the CuWO_4_ nanorod arrays of 50 nm and around 620 nm for gaps between the CuWO_4_ nanorod arrays of 150 nm, which shows that the absorption originates from the CuWO_4_ material.

To test the maximum carrier generation and the minimum carrier recombination of the CuWO_4_ nanorod arrays on all the samples with distinct gaps between the CuWO_4_ nanorod arrays, the PEC measurement was carried out on the photoanode of the CuWO_4_ nanorod array and is shown in Figure 4c. The working electrode measurement of the CuWO_4_ nanorod arrays was carried out using three electrodes with an Ag/AgCl electrode reference and a Pt plate as the counter electrode. All the measurements were performed in 0.1 M Na_2_SO_4_ (pH 6.8). This procedure was conducted to minimalize the effects of outside bubbles that may occur during the measurement due to irradiation using a solar simulator.

**Figure 5:**
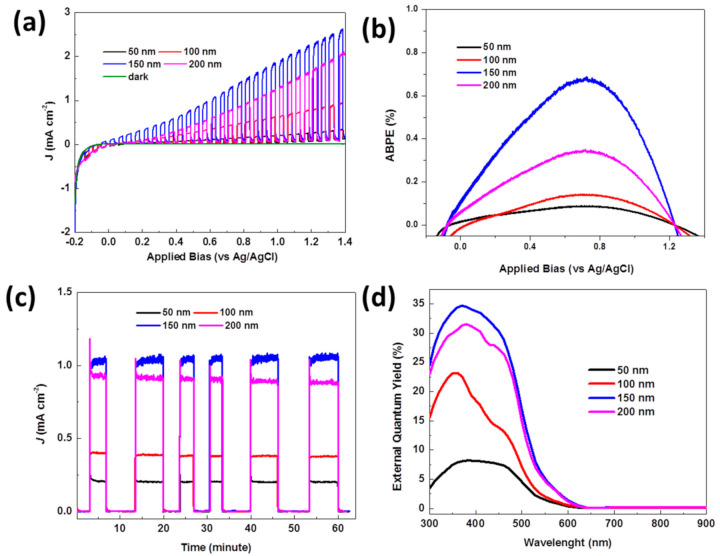
(**a**) Illumination of on–off rays with a solar simulator and irradiation of 100 mW cm^−2^ with a typical LSV (*J-V*) on the photoanode of the CuWO_4_ nanorod arrays using three electrodes. The scanning was set with a bias potential in the range of −0.2 up to 1.4 V vs. Ag/AgCl; (**b**) APBE calculations of each of the samples of the photoanode based on the data shown in (a). (**c**) the stability measurement of the photoanode of the CuWO_4_ nanorod arrays (*J-t*) that was set with a constant potential of 0.7 V vs. Ag/AgCl using a solar simulator and irradiation of 100 mW cm^−2^ for as long as 1 h; and (**d**) the EQY measurements for each of the photoanode of the CuWO_4_ nanorod arrays. All the measurements in Figure 5 were carried out by using an electrolyte of 0.1 M sodium sulfate.

Figure 5a shows the characteristics of current density from irradiation on the photoanode of the CuWO_4_ nanorod arrays versus the bias potential interval (*J-V*) that is measured at a potential rate of 20 mV s^−1^ and a potential range between −0.2 V to 1.4 V vs. Ag/AgCl in dark condition (shown by the green line) and an irradiation of AM 1.5 G, 100 mW cm^−2^ on the photoanode of the CuWO_4_ nanorod arrays. All the samples with distinct in-between gaps and diameters (shown in the SEM image in Figure 2) were tested using the previously described measurement. The images clearly show an increase in irradiation current in areas with a positive bias voltage, which shows that CuWO_4_ nanorod arrays are type-n semiconductors. For comparison, in the images given in Figure S2 of the Supplementary Materials, all the measured samples of the CuWO_4_ nanorod arrays show the same working area, which is in the positive voltage area. The fundamental difference that can be observed from this measurement is the magnitude of irradiation current density at intervals between 0 V and 0.4 V vs. Ag/AgCl. As described in previous research, there exists a possibility that a transient effect occurs between the standing current and membrane caused by an imperfect bonding between the particles of CuWO_4_ that causes a decrease in the current density of the irradiation. Therefore, adding a very thin layer of TiO_2_, approximately around ±5 nm (*E_gap_* TiO_2_ 3.2 eV) using ALD on the surface of the CuWO_4_ nanorod arrays, may increase the capacity of the light absorption spectrum of the CuWO_4_ nanorod arrays. This is despite previous research findings, in which the photoanode of CuWO_4_ obtained a maximum value of 3% to change the incoming light irradiation to hydrogen due to the large energy gap between the valence band and the conduction band. Nevertheless, the presence of the Au particles from the beginning of the fabrication process of the imprinted AAO template can also cause a surface plasmon resonance (SPR) effect. Hales et al. stated that the magnitude of the SPR effect depends on the size, morphology, and density of the Au particles [23]. The Au particles that are scattered on the surface of the photoanode are also used as a conductor for the deposition of the CuWO_4_ nanorod arrays in the early phase, as mentioned in the steps in Figure 1. For comparison, Figure S2 of the Supplementary Materials shows the photogenerated measurement on CuWO_4_ thin films. CuWO_4_ thin films were deposited on FTO glass with the same mechanism as the fabrication method of the CuWO_4_ nanorod arrays. Likewise, the photogenerated measurement on CuWO_4_ thin films used the same electrolyte and bias voltage limit range. It is clearly shown in Figure S2 of the Supplementary Materials that the maximum irradiation current density of the applied bias potential thermodynamics is around 0.74 mA cm^−2^, which is lower than the same measurement performed on the compartments of the CuWO_4_ nanorod arrays. The logical reasoning behind this is that hole injection on CuWO_4_ thin films to slow electrolytes and the weak current carrier enables the CuWO_4_ thin films to have a lower performance toward its PEC water splitting measurement.

Figure 4 nanorod arrays using the equation, which is *η* = *J*(1.23 − *V_app_*)/*P_light_*, where *V_app_* applies a bias potential vs. Ag/AgCl, *J* is the external current density, and *P_light_* is the irradiation power. The results of the measurement are presented in Figure 5b, showing a measurement of 0.06% (at 0.681 V vs. Ag/AgCl), 0.12% (at 0.683 V vs. Ag/AgCl), 0.72% (at 0.712 V vs. Ag/AgCl), and 0.38% (at 0.711 V vs. Ag/AgCl) for gaps between the CuWO_4_ nanorod arrays of 50 nm, 100 nm, 150 nm, and 200 nm, respectively. From the measurements presented in Figure 5b, it can be seen that the optimal electrode gap between the CuWO_4_ nanorod arrays is 150 nm. As a result, the composition of the CuWO_4_ nanorod arrays has a wider layer and is in direct contact with the electrolyte and, in turn, serves as an effective conductor of electrons from the interface of the working electrode to the counter electrode and vice versa, causing a quick charge transfer. Nevertheless, a gap too close or too far between two diamagnetic CuWO_4_ nanorod arrays can significantly affect the created electric field [24]. As a result, an optimal gap can have a significant effect on this diamagnetic material. Qualitatively, it can be interpreted that polarization of the induction current and the noncarrier of the magnetic field orbit can efficiently influence the irradiation conversion. As shown in Figure 5c, the characteristics of AM 1.5G irradiation on the CuWO_4_ nanorod arrays seem relatively stable within 60 min and in a dark–light condition. When the current resulted from irradiation reaches a value of 1.02 mA cm^−2^ with 0.7 V vs. Ag/AgCl, this result is a significant improvement, compared to previous research. For comparison, we show the measurement of current irradiation of CuWO_4_ thin films in Figure S3 of the Supplementary Materials. This verifies our hypothesis that the photoanode of the CuWO_4_ nanorod arrays significantly influences photogeneration, which is shown by CuWO_4_ nanorod arrays exhibiting an absorption and photon collection through the bandgap transition between electrodes. The highest value comes from the electrode with an in-between gap of 150 nm, corresponding to the highest absorption curve shown in Figure 4b.

As for the EQY (%) measurement on the photoanode of the CuWO_4_ nanorod arrays for every sample, the measurement is carried out without the use of bias voltage, as shown in Figure 5d. The EQY peak occurs at wavelengths around 410 nm. For additional information, this photon-to-current peak efficiency occurs for all the samples with distinct gaps between the CuWO_4_ nanorod arrays. As stated before, the effect of the gap between the CuWO_4_ nanorod arrays is very transparent. A close gap between the CuWO_4_ nanorod arrays significantly influences a decrease in the efficiency value of the measurement. As a result, photon absorption of electrodes is effective within the relatively wide wavelength interval of visible light, which is in the range of 300 nm up to 620 nm. This is proof that nanostructures can significantly influence an increased response toward incoming light at wavelengths between 300 nm and 620 nm. The effect of the gap between the CuWO_4_ nanorod arrays significantly impacts the performance of this photoelectrode.

**Figure 6:**
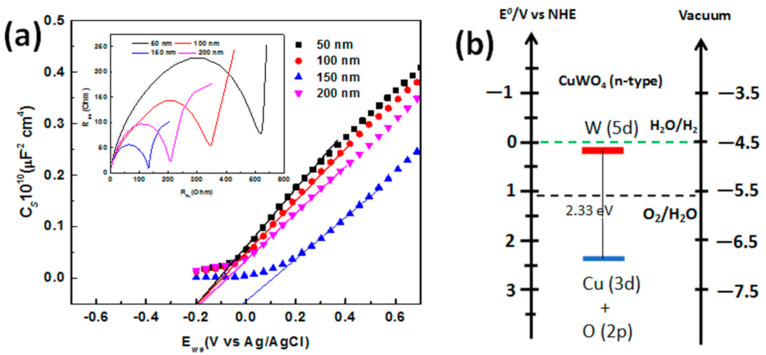
(**a**) The Mott‒Schottky plot used to determine the direct voltage of the flat band for each of the photoanode of the CuWO_4_ nanorod arrays (the smaller image shows the SPEIS measurement for the Nyquist plot) and (**b**) the energy level scheme of the photoanode of the CuWO_4_ nanorod arrays.

Next, a measurement to describe the electronic structure of the CuWO_4_ nanorod arrays composite is needed. For this, a relevant Mott–Schottky (M–S) plot is used and also by observing the electrochemical impedance, which, in this research, starts from a frequency of 1 Hz up to 1 MHz with a bias potential interval of −0.4 V up to +0.7 V vs. Ag/AgCl. The capacitance value of the measurement is presented in Figure 6a at a frequency of 1 kHz. From this, it is shown that the M–S plot from each electrode with a distinct gap between the CuWO_4_ nanorod arrays in the PEC water splitting procedure has a linear profile and a positive slope that demonstrates features of a type-n semiconductor. These data are then used for the M–S relation, in which the typical potential of the flat band V*_FB_* is in the range of 0.2 V vs. Ag/AgCl up to 0.19 V vs. Ag/AgCl, which is in reference to previous research [24,25]. From the results, we obtained a plot and is linearly projected on each distinct flat bond potential of −0.16 V (vs. Ag/AgCl), −0.14 V (vs. Ag/AgCl), −0.104 V (vs. Ag/AgCl), and 0.46 V (vs. Ag/AgCl) in the open-circuit voltage under 100 mW cm^−2^ irradiation for each electrode with distinct gaps between the CuWO_4_ nanorod arrays of 50 nm, 100 nm, 150, and 200 nm, respectively. Then, the density of the majority charge carrier is calculated from the gradient of the M–S plot by using the equation d(*C_sc_*^−2^)/dE = 2/*eε_o_εN_D_*, where the dielectric constant is 83 for the CuWO_4_ nanorod arrays and the effective electron mass is 70 [24,26]. From the calculation, the density of the majority charge carrier of 0.84 × 10^20^ cm^−3^, 0.97 × 10^20^ cm^−3^, 2.86 × 10^20^ cm^−3^, and 1.35 × 10^20^ cm^−3^ are obtained for distinct gaps between the CuWO_4_ nanorod arrays of 50 nm, 100 nm, 150, and 200 nm, respectively. The results show a higher majority charge carrier than that reported in previous research [24,26]. Figure 6a also clearly demonstrates that the CuWO_4_ nanorod arrays have the behavior and conductivity of a type-n semiconductor, and the highest value of the majority charge carrier belongs to the CuWO_4_ nanorod array with an in-between gap of 150 nm. As shown in the inset of Figure 6a, the Nyquist plot is measured in the frequency range of 10^6^ Hz up to 1 MHz with a bias of 0 V vs. Ag/AgCl for two sets of electrodes in PEC. In addition, this is to show that a Nyquist plot resulting in a half circle at the highest frequency is the fundamental characteristic of the process of charge transfer throughout the process of measurement. Furthermore, the diameter of the half circle formed by the Nyquist plot is relatively equal to the charge transfer resistance [27]. The overall electrochemical measurements show that the modification of the CuWO_4_ nanorod arrays has a positive impact on the electronic nature of the CuWO_4_, which is indicated by the increase in doping density and area of band banding on the interface of the photoanode and electrolyte. Additionally, an observation on charge transfer resistance can be made on the overall electrochemical measurements, that is, the smaller the resistance, the more useful it is to promote charge separation in water molecules and charge carrier transfer as a result of irradiation. This provides a higher efficiency signal for utilizing charge generated from photogeneration of the CuWO_4_ nanorod arrays, compared to that of CuWO_4_ thin films.

On the other hand, determining the proper bandgap of the CuWO_4_ nanorod arrays based on bandgap values obtained using the Tauc plot method is shown in Figure 6b. It is known that a water splitting reaction must be at the right range of standard reduction and oxidation potential because CuWO_4_ nanorod arrays have valence bands and conduction bands within that range, thus making CuWO_4_ nanorod arrays a very good material for the PEC water splitting procedure. To summarize, the small distance between the Fermi level and the valence band indicates that CuWO_4_ nanorod arrays are the desired type-n semiconductor. The estimated bandgap has a slightly lower value than that of previous research that is closely related to material defect [13], in which the energy was obtained in vacuum conditions, and surface defect was disregarded through an ion milling procedure. This procedure causes a significant difference in bandgap that is measured with the electrochemical technique, compared to the procedure that we carried out.

**Figure 7:**
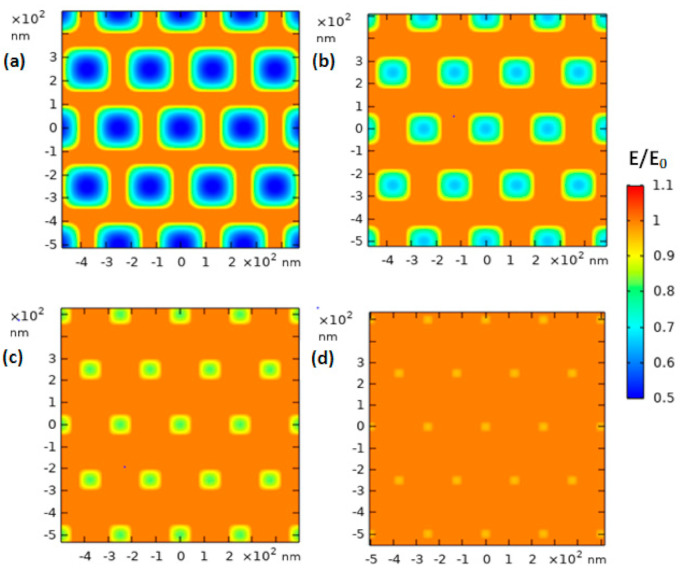
A top view of the FDTD simulation of the photoanode of the CuWO_4_ nanorod arrays toward EM waves with a wavelength of 420 nm for each of the samples of the photoanode of the CuWO_4_ nanorod arrays with distinct gaps between the CuWO_4_ nanorod arrays of (**a**) 50 nm, (**b**) 100 nm, (**c**) 150 nm, and (**d**) 200 nm.

We carried out a simulation of the selected material to further understand the interaction between the photon and the CuWO_4_ nanorod arrays using the FDTD method and is shown in Figure 7 for each distinct gap between the CuWO_4_ nanorod arrays. From the outcome of this simulation, information on the electric field distribution of photon irradiation with a specific wavelength of 410 nm on areas of the CuWO_4_ nanorod arrays is obtained and captured from the top layer. The distinct gaps between the CuWO_4_ nanorod arrays observed ranged from small to large gaps. The density of electric field coupling on all types of material can produce a strong energy contour, thus having a big influence on the efficiency signal of spectroscopy. As a result, the distribution of the CuWO_4_ nanorod arrays produced by the scattering of the nanorod arrays is in line with nano metal particle arrays. Jain et al. [28] explained that bipolar coupling caused by the in-between gaps will result in a decay of the plasmon coupling, and this will become irrelevant on metal, material form, and the dielectric constant of all types of material. Moreover, the increase of intensity in the near field indicates a faster decay as the gap of the CuWO_4_ nanorod arrays becomes bigger. In other words, if the gaps between the CuWO_4_ nanorod arrays are made to be bigger, then the electric field coupling becomes weaker. This weakening of the coupling may be one of the reasons a bandgap shift occurs in the CuWO_4_ nanorod arrays. However, the bandgap shift that occurs in the CuWO_4_ nanorod arrays can be controlled by setting the gaps between the CuWO_4_ nanorod arrays of the samples to 150 nm and 200 nm based on the conventional quantum confinement effect. In other words, the results give information that a bandgap shift can be achieved by controlling the diameter and the in-between gaps on the conventional quantum confinement effect even though the obtained parameters are relatively bigger than that of the Bohr radius. Due to the reasons above, along with the relative energy density of the CuWO_4_ nanorod arrays, which is not significantly different, the quantum effect shows that a cumulative change in thickness of the CuWO_4_ nanorod arrays is not accounted for. However, the bandgap shift is specifically tied to the interaction between the CuWO_4_ nanorod arrays after irradiation and the uniformity of the structure of the material. The stability of the bandgap is in relevance to the power of the near-field light, where this is used to increase the advantages of the optical properties of the CuWO_4_ nanorod arrays to be used as material for the application of photoelectric.

The FDTD simulation on the CuWO_4_ nanorod arrays gives information on the interaction connection between the photon and the geometric parameter of the CuWO_4_ nanorod arrays that possess distinct gaps between the arrays, thus resulting in optimal electric field intensity. The simulation that was taken from irradiation on the top layer of the CuWO_4_ nanorod arrays shows the abundant distribution of counter anode along the layer that differentiates CuWO_4_ nanorod arrays with the medium. It can be seen in Figure 7 (also see Figure S3 of the Supplementary Materials) that as the gaps between the CuWO_4_ nanorod arrays become bigger, the electric field becomes weaker; both of them were carried out at close to 700 nm. Likewise, as the gaps between the CuWO_4_ nanorod arrays become smaller, the electric field becomes stronger. Nevertheless, the electric field that occurs on the CuWO_4_ nanorod arrays also affects the magnetic field; thus, it has relevance toward the penetration of the electron charge of electrolytes on the CuWO_4_ nanorod arrays. As a result, a suitable gap between the CuWO_4_ nanorod arrays is needed to obtain a magnetic field and an electric field that significantly contributes toward the performance of the PEC water splitting procedure. The results of this experiment and simulation clearly show that CuWO_4_ nanorod arrays greatly contribute toward the efficiency of CuWO_4_ thin films.

## 4. Conclusions

In conclusion, to the best of our knowledge, this is the first time that the fabrication of CuWO_4_ nanorod arrays using imprinted AAO templates by electrodeposition was carried out. The photoanode of the CuWO_4_ nanorod arrays was fabricated with four distinct gap variations and is what makes it superior to other compartments such as CuWO_4_ thin films. It is proven that with this method, the fabricated CuWO_4_ nanorod arrays show a high degree of material purity when tested using XRD and XPS. Additionally, when observed with SEM and EDX, it is proven that the CuWO_4_ nanorod arrays material structure can be controlled and also produce a chemical structure from its three-molecule compound composition. A realization of CuWO_4_ nanorod arrays that have a homogenous structure and have suitable gaps between the CuWO_4_ nanorod arrays is key to optimizing the penetration of charge between electrolyte and photoanode to absorb incoming photons from irradiation of 100 mW cm^−2^. The maximum photogeneration of the CuWO_4_ nanorod arrays was obtained at a gap between the CuWO_4_ nanorod arrays of 150 nm and a current density of 1.01 mA cm^−2^, which resulted in an APBE peak at a bias voltage of 0.7 V vs. Ag/AgCl. The results of this research are further strengthened by an FDTD simulation that generated magnetic field energy and electric field energy that match the experiment that was carried out, in which the peak energy was contrary to its absorbance at a wavelength of around 700 nm.

## Data Availability

Not applicable.

## Supplementary Materials

The following are available online, Figure S1: XPS survey of CuWO_4_, core-level XPS of Cu2p, core-level XPS of W 5d, and core-level XPS of O1s, respectively, Figure S2: (a) LSV of a CuWO_4_ thin film photoanode in a three-electrode configuration described in the text and under AM 1.5 G sunlight. The scans are collected from −0.2 to 1.4 V versus Ag/AgCl. (b) ABPEs of the relevant electrodes from (a), Figure S3: Electric energy and magnetic energy from FDTD simulation by CuWO_4_ nanorod arrays in the in-between gap, Table S1: Previous report for photoelectrochemical measurements of the kinds CuWO_4_ photoanode for oxygen evolution reaction.
