# Supplementary material for: Tunning of Templated CuWO4 Nanorods Arrays Thickness to Improve Photoanode Water Splitting
Source: Molecules. 2021 May 13;26(10):2900. doi: 10.3390/molecules26102900 (PMC8153333; doi:10.3390/molecules26102900)
Supplement: Supplementary file 1 [file molecules-26-02900-s001.zip › molecules-1156944-supplementary.pdf]

Supplementary Materials

Tunning of Templated $CuWO_4$ Nanorods Arrays Thickness to Improve Photoanode Water Splitting

Nasori Nasori [1,2,*], Dawei Cao [3], Zhijie Wang [4], Ulya Farahdina [2], Agus Rubiyanto [2], and Yong Lei [1,*]

[1] Group of Applied Nanophysics (Fachgebiet Angewandte Nanophysik), Institute of Physics & IMN MacroNano® (ZIK Technical University of Ilmenau, 98693 Ilmenau, Germany; yong.lei@tu-ilmenau.de (Y.L.)

[2] Department of Physics, Faculty of Sciences, Sepuluh Nopember Technology Institute, Surabaya 60111, Indonesia; kanganas01@gmail.com (N.N.); ulyafarahdina06@gmail.com (U.F); agus.rubi65@gmail.com (A.R.)

[3] Department of Physics, Faculty of Sciences, University of Jiangsu, Zhenjiang 212013, China; dwcao@ujs.edu.cn (D.C.)

[4] Semiconductor Materials Science Key Laboratory, Semiconductors Institute, Chinese Sciences Academy, Beijing 100083, China; wangzj@semi.ac.cn (Z.W.)

* Correspondence: nat.nasori@physics.its.ac.id (N.N.); yong.lei@tu-ilmenau.de (Y.L.)

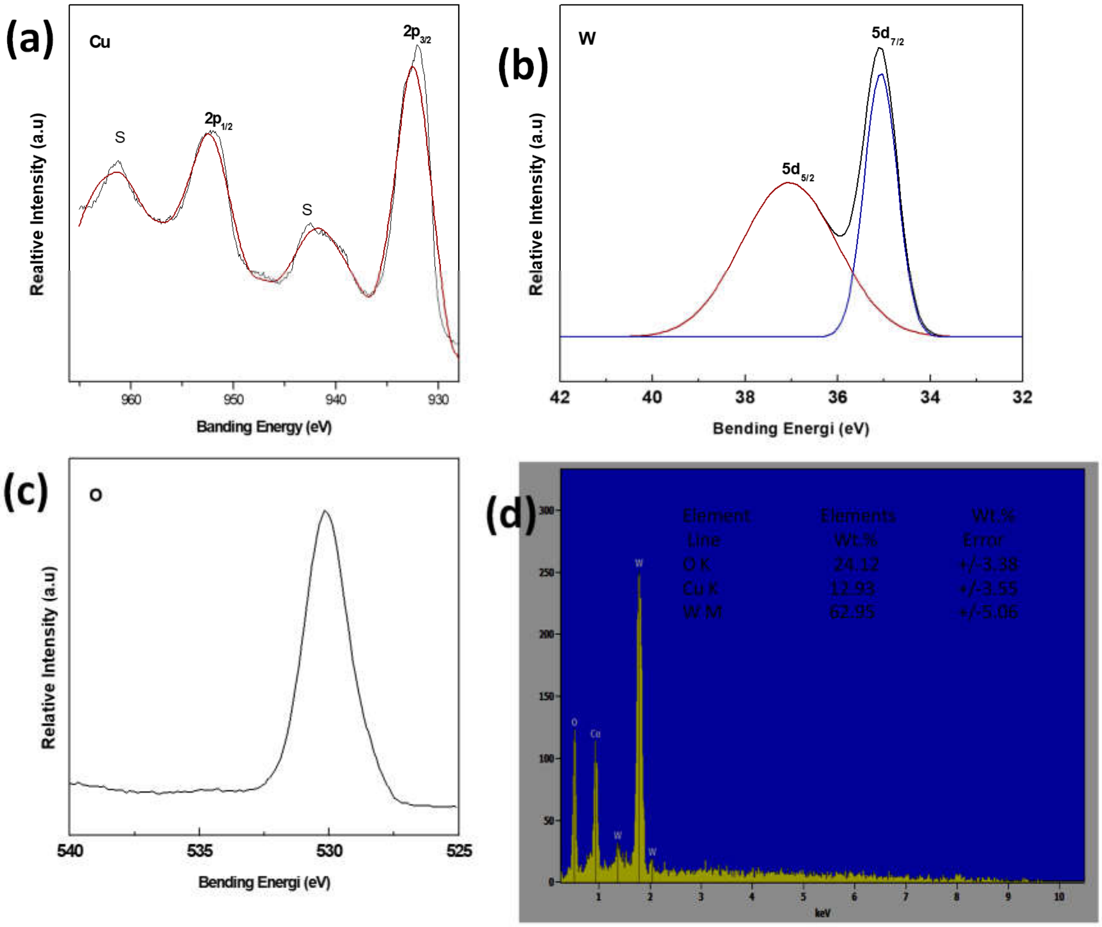

Figure S1 XPS survey of $CuWO_4$, core-level XPS of of Cu2p, core-level XPS of W 5d, and core-level XPS of O1s, respectively.

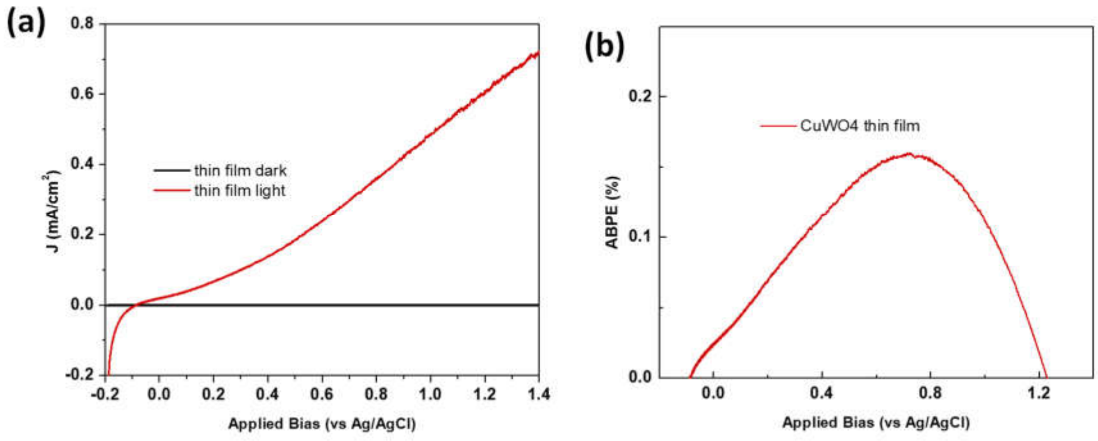

Figure S2 (a) LSV of a $CuWO_4$ thin film photoanode in a three-electrode configuration described in the text and under AM 1.5G sunlight. The scans are collected from −0.2 to 1.4 V versus Ag/AgCl. (b) ABPEs of the relevant electrodes from (a).

Table S1 Tabel Previous report for photoelectrochemical measurements of the kinds $CuWO_4$ photoanode for oxygen evolution reaction

Photoanode Speciment	Solution as Electrolyte		Linear potential sweep measurements		Constant potential measurements	
			**Scan rate (mV s^{-1})**	**J_{ph} mA cm^{-2}) at 1.57 RHE**	**Potensial (V vs RHE)**	**J_{ph} (mA cm^{-2}) after 1 h**
**This work:**						
**CuWO4**	0.1 M Na_2SO_4	pH 6.8	10	0.3	1.21	0.26
**CuWO4 nanorod**	0.1 M Na_2SO_4	pH 6.8	10	1.01	1.21	0.98
**$CuWO_4$ nanoflake [1]**	0.1 M borate buffer	pH 9	10	0.58	1.57	0.52
**H-treated**	0.1 M phosphate buffer	pH 7	10	0.58	-	-

**$CuWO_4$ nanoflake** [1]	0.1 M borate buffer	pH 9	10	0.84	1.57	0.69
	1 M phosphate buffer	pH 7	10	0.82	-	-
**$CuWO_4$** [2]	0.1 M borate buffer	pH 9	10	0.22	-	-
	0.1 M phosphate buffer	pH 7	10	0.2	-	-
	0.05 M H_2SO_4	pH 1	10	0.15	-	-
**$CuWO_4$** [2]	0.1 M phosphate buffer	pH 7	20	0.26	1.23	0.064
	0.1 M phosphate buffer	pH 5	-	-	1.23	0.072
	0.1 M borate buffer	pH 7	-	-	1.23	0.075
	0.1 M phosphate buffer	pH 3	-	-	1.23	0.062
**$CuWO_4$** [2]	0.1 M phosphate buffer	pH 7	10	0.26	1.13	0.07
	0.1 M acetate buffer	pH 2.9	10	0.23	0.874	0.038
	0.1 M $HClO_4$	pH 1.2	10	0.22	0.772	0.003
**$CuWO_4$** [3]	0.5 M borate buffer + 0.2 M KCl	pH 7	50	0.14	-	-
**$CuWO_4$** [3]	0.1 M borate buffer + 0.2 M KCl	pH 9	20	0.15	1.23	0.09
**$CuWO_4$** [3]	0.1 M phosphate buffer	pH 7	-	0.04	1.23	0.02
**$CuWO_4$** [2]	0.33 M H_3PO_4	pH 0	25	0.36	-	-

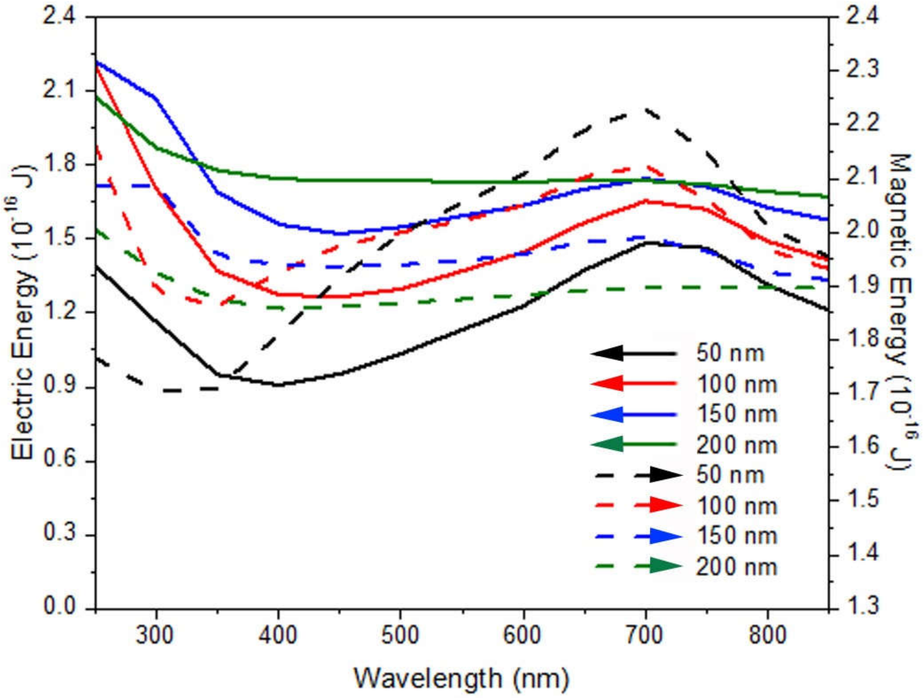

Figure S3 Electric energy and magnetic energy from FDTD simulation by $CuWO_4$ nanorod arrays in in-between space.

Reference

1. Yourey, J.E.; Pyper, K.J.; Kurtz, J.B.; Bartlett, B.M. Chemical Stability of $CuWO_4$ for Photoelectrochemical Water Oxidation. *The Journal of Physical Chemistry C* **2013**, *117*, 8708–8718.
2. Yourey, J.E.; Bartlett, B.M. Electrochemical Deposition and Photoelectrochemistry of $CuWO_4$, a Promising Photoanode for Water Oxidation. *J. Mater. Chem.* **2011**, *21*, 7651–7660, doi:10.1039/C1JM11259G
3. Gaillard, N.; Chang, Y.; DeAngelis, A.; Higgins, S.; Braun, A. A Nanocomposite Photoelectrode Made of 2.2 EV Band Gap Copper Tungstate ($CuWO_4$) and Multi-Wall Carbon Nanotubes for Solar-Assisted Water Splitting. *International Journal of Hydrogen Energy* **2013**, *38*, 3166–3176, doi:10.1016/j.ijhydene.2012.12.104.
